# A Speech-Segregation Algorithm for Spatial Hearing Aids to Operate With Multiple Sound Sources

**DOI:** 10.1044/2025_JSLHR-25-00648

**Published:** 2026-02-23

**Authors:** Jakeh E. Orr, Atra Z. Eslami Boudreaux, Irmak Gokcen, Yan Gai

**Affiliations:** a Biomedical Engineering Department, School of Science and Engineering, Saint Louis University, MO

## Abstract

**Purpose:**

Hearing-aid users often face challenges in noisy environments due to time, level, and spectral cues being compromised by current generation hearing aids. This study explored a physiologically based speech-segregation algorithm that selectively removes or attenuates unwanted sound sources.

**Method:**

In our previously developed localization algorithm, the time-frequency responses after a unique normalization approach always reside inside the unit circle of the model space. Given the “sparseness” property of daily sound, the model forms distinct clusters that correspond to the source locations. In the present study, the localization model was adapted to segregate speech by using a binary mask to remove the cluster of unwanted sound. The speech target was one of 200 intelligible sentences. The interfering sound was time-reversed sentences in a random sequence spoken by the same speaker. Automatic speech recognition transcribed the sound mixture before and after the segregation algorithm.

**Results:**

When both target and noise were located at the front, applying a hard mask (i.e., 1 or 0) almost perfectly removed the energy of noise. When the sound sources moved to the side or back with smaller angular separations, clusters were less distinguishable, leading to worse intelligibility performance. Applying a soft mask (i.e., 1 or 0.2) instead showed slightly lower performance for the front but improved performance for the back and side.

**Conclusion:**

Our algorithm performs localization and segregation in a combined and straightforward manner, potentially for spatial hearing aids to function better in challenging listening environments.

Hearing-aid users, even with a single monaural device, can generally achieve decent speech intelligibility when listening in a quiet environment. However, they often struggle to discriminate sound sources when there are multiple talkers or in a noisy environment, significantly limiting their communication effectiveness. Normal-hearing listeners' binaural systems not only localize sound sources but also segregate them with selective attention (Saberi et al., 1991). Unfortunately, spatial cues are highly compromised with conventional hearing aids. It has been shown that speech reception thresholds in noise are worse for normal-hearing listeners when wearing hearing aids due to compromised spatial perception and speech intelligibility (Cubick et al., 2018). The above examples point to the issue lying within the hearing aid directly, not as an inherent problem of hearing loss.

Modern hearing aids strive to provide users with restored spatial sensitivity and release from masking. Directional microphones have been proven to bring benefits by increasing the signal-to-noise ratios (SNRs; Jespersen et al., 2021; Ricketts, 2005). However, studies have found that listeners using directional microphones experience substantial changes in signal intelligibility or detection depending on the angle between listener and signal source. For example, Brimijoin et al. (2014) showed that when the angle becomes large (i.e. listener turns away from “on-axis”), performance drops substantially. Another promising strategy called binaural beamforming combines signals from both sides with up to four microphones, leading to large SNR improvements (Best et al., 2015; Derleth et al., 2021; Srinivasan et al., 2008). Unfortunately, binaural cues are often compromised in this process, and spatial awareness is thus reduced.

The present study explored a physiologically based audio-processing algorithm that integrates the localization and segregation of multiple concurrent spatial sound. Our strategy falls in the general category of blind speech separation. Blind speech separation can be divided into three general categories of approaches (Makino et al., 2007). When it is performed with multiple microphones and the number of sources is no more than the number of microphones, the mechanism is called “determined” separations. A major approach in this category is based on the independent-component analysis (ICA) or its variants. When sound sources are independent, the ICA can sometimes accurately or even perfectly uncover the hidden sources. A commonly used ICA-based algorithm is the Joint Approximate Diagonalization of Eigenmatrices (JADE) approach (Rutledge & Jouan-Rimbaud Bouveresse, 2013). Based on our experience, successful analysis using the JADE relies on iterative computations and may be more time consuming for real-time processing than the algorithm presented in this study. The JADE also has the limitation that the number of sources cannot be more than the number of signal channels, for example, microphones (Semmlow & Griffel, 2014).

If there are more sources than microphones, which is common for hearing-aid users, the segregation process is called “underdetermined” (Makino et al., 2007). With two microphones (i.e., one for each ear), as is in the case for in-the-canal hearing aids, a successful and relatively simple approach for underdetermined blind-source separation relies on the *sparseness* property of sound signals. Here, sparseness refers to the fact that natural sound, as opposed to broadband noise, is usually limited with their frequency components. It is common for our daily-life sound sources to overlap in the time domain (i.e., concurrent). However, when projected into a two-dimensional time-frequency domain, each time and frequency spot is rarely occupied by multiple talkers due to different voice pitches and spoken contents. Adding the frequency dimension is the key to creating the desired sparseness property.

With the sparseness property, a K-means clustering approach can be applied to datapoints derived with short-time Fourier transform (STFT; Makino et al., 2007). This is indeed what was utilized in our previous localization model (Orr et al., 2023) with a special normalization process (Keyrouz, 2017) to form spiral-shaped cluster trajectories. In the present study, the localization model was extended to a speech-segregation model by adding a binary mask. The mask can selectively remove or retain certain time-frequency points in the spectrogram of a sound. Note that there can be a small percentage of misbehaviors when certain datapoints contain both sounds.

The third category of blind-speech segregation uses a single microphone, called monaural source separation (Makino et al., 2007). Major strategies include (a) top-down generative models based on probabilistic decompositions of sound spectra or other statistics (Hershey et al., 2010; Li et al., 2010), and (b) the computational auditory scene analysis (CASA; Cooke et al., 2010). CASA does not necessarily use spatial cues to separate auditory objects; it can utilize features such as temporal coherence or frequency spectra (Shamma et al., 2011). The monaural algorithms are frequently used in improving automatic speech recognition (ASR) software (Cooke, 2006; Shao et al., 2010).

In recent years, machine learning, especially deep-learning neural networks, has been adopted in blind-speech–segregation algorithms (Awotunde et al., 2020; Bentsen et al., 2018). These are relatively resource-intensive software solutions that may prove to be difficult when applied in real-time sound processing.

Considering the three important sound-localization cues used by the human auditory system, namely, the interaural time difference (ITD), interaural level difference (ILD), and monaural spectral cues (Loiselle et al., 2016; Tollin & Yin, 2009), the exact compromise in those cues may depend on the type of hearing aids. The duplex theory (Mills, 1972; Tollin & Yin, 2009) states that, at low sound frequencies (e.g., < 2 kHz), sound localization in azimuth relies on ITDs and at high frequencies (e.g., > 2 kHz), ILDs dominate the localization. ITDs are time delays between sound arrivals at the two ears; they are mostly lost in bilateral (i.e., with two independent speech processors) hearing aids and somewhat preserved in binaural (i.e., with one coordinated speech processor) hearing aids. Also, as sound travels, its strength dissipates (Zahorik & Kelly, 2007). ILDs are created by the head and pinna shadows. ILDs are relatively easier to preserve, since accurate timing is not required between the two hearing devices.

Localization in elevation relies on monaural spectral cues, which are contained in a pair of head-related transfer functions (HRTFs), one for each ear. HRTFs change with the spatial location and are affected by the shape of the outer ear (pinna), adding spectral cues for sound signals based on their arrival directions. ITD and ILD cues are also contained in the HRTFs. To best preserve the monaural spectral cues, microphones need to reside inside the ear canals.

Utilizing the sparseness property, we previously developed a sound-localization model (Orr et al., 2023). Because each sound source has been encoded with the HRTF convolution, the model forms distinct clusters after STFT and a normalization procedure (Keyrouz, 2017). In that model, when the sound location moves from left to right, the clusters form a spiral pattern at a fixed model frequency (Orr et al., 2023). That model can localize multiple sound sources simultaneously. In fact, if only the horizontal location is considered, one model frequency is sufficient. When both the horizontal and vertical locations are considered, the model needs to be examined at multiple frequencies, essentially tracking the HRTFs (Keyrouz, 2017).

The present study is built on similar mechanisms as the sound-localization model but extends the model for speech segregation. Speech sentences and/or speechlike sound carried spatial information using the virtual-acoustic–space technique (Hartmann & Wittenberg, 1996). HRTFs of three human subjects were obtained from the Princeton 3D3A database (Sridhar et al., 2017). After STFT and the normalization procedure, a spiral model was generated at every frequency, covering the entire frequency range of the STFT. Because we know exactly which cluster corresponds to which spatial location, removing a cluster will likely remove the energy for that unwanted sound source. The removal process was performed by applying a time-frequency binary mask (Makino et al., 2007). The resulting time-frequency responses were then converted to the time domain and transcribed by an ASR software to obtain a speech-recognition score. Previous studies have shown high correlations (i.e., > 90%) between ASR-based algorithms and human performance for English (Karbasi & Kolossa, 2022) and non-English (Schädler et al., 2015) languages. In the present study, if the segregation algorithm is successful, this score should be higher than the score achieved when the raw mixture of speech and interference is transcribed by the ASR.

In summary, the present study aims to reconstruct a localization model into a spatial-segregation model by utilizing physiologically based spatial cues and constructing time-frequency masks to segregate speech signals. The present study and our previous work (Orr et al., 2023) intend to provide solutions that conquer the cocktail-party challenge.

## Method

Here, we will present the approaches for measuring speech intelligibility and constructing the segregation algorithm. Since ASR was used and no human subjects were tested, the study was not subject to or exempt from an approval process.

### Speech and Time-Reversed Sentences

In this study, two concurrent spatial sound sources were simulated using the virtual-acoustic–space technique. Both the speech target and interfering sound were constructed from a pool of sentences created by Calandruccio and Smiljanic (2012). In each sentence, there are exactly four key words. Each group of sentences contains 25 sentences, forming a pool of 100 key words. Example sentences are “The PARK OPENS in ELEVEN MONTHS” and “The THIRSTY KID DRINKS JUICE.”

In the present study, on each trial, the speech target was one of 200 intelligible sentences (eight groups) spoken by a female speaker. The continuous noise stream was time-reversed sentences, arranged in a random sequence, spoken by the same female speaker. Using time-reversed sentences preserves most of the human speech features while preventing the interfering sound to be transcribed by the ASR, as the software itself may not have distinguished targets from maskers if both were intelligible sentences. For most of the conditions, both the target and interfering sound had a virtual sound intensity of 75 dB SPL on average, yielding an SNR of 0 dB. The exact sound intensity should not matter to the ASR performance, as no real human ear or nonlinear auditory model was involved. The 0 dB SNR allows us to examine difficult listening conditions. For the last condition, SNR was increased to 3 dB.

**Figure 1.**
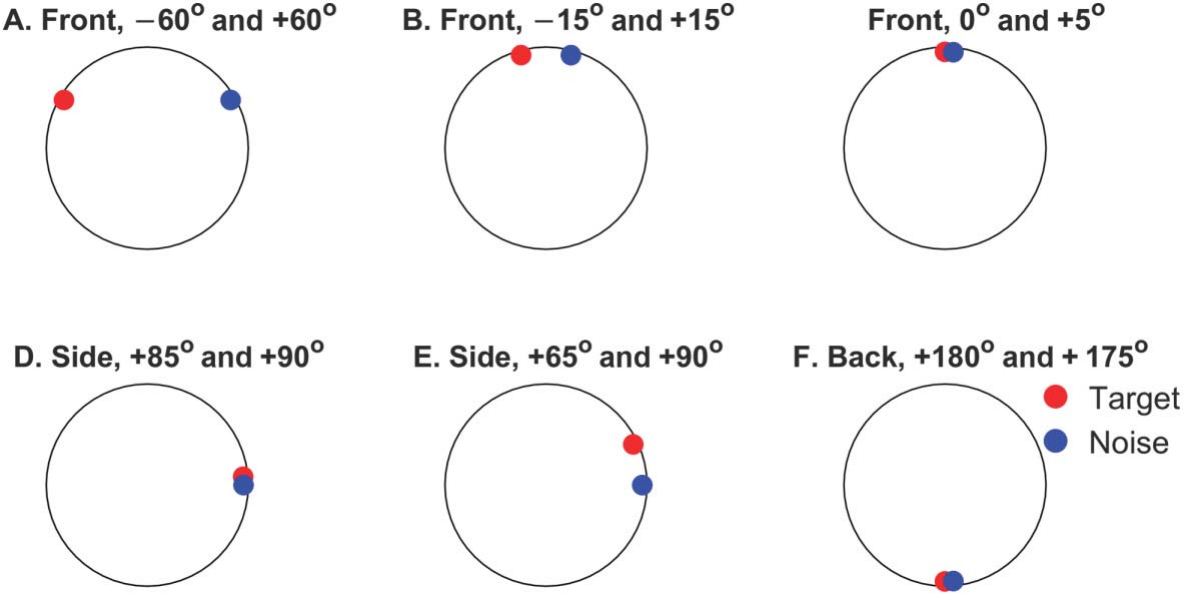
Spatial configurations of the target (red) and interfering-noise (blue) locations for the six virtual-space conditions. The center of the circles is where the virtual location of the ears is supposed to be.

HRTFs obtained from three human subjects were selected from the 3D3A database (Subjects 1, 15, and 20 denoted in this study as HRTFs-1, HRTFs-2, and HRTFs-3). Since the ASR performance was similar across the three HRTFs, we did not test more HRTFs from the databases. Six conditions of spatial placements of the target and interfering sound were tested, as shown in Figure 1. Three conditions placed the target and interfering sound at the front with spatial separations of 120° (see Figure 1A, −60° and +60°), 30° (see Figure 1B, −15° and +15°), and 5° (see Figure 1C, 0° and +5°). Two conditions placed the sound on the right side with separations of 5° (see Figure 1D, +85° and +90°), and 15° (see Figure 1E, +65° and +90°). The last condition tested the back locations with a separation of 5° (see Figure 1F, +180° and +175°).

After determining the target and interfering-sound locations, the speech sentence and the time-reversed sentence were individually convolved with the head-related impulse responses (HRIRs) for the corresponding locations. The two stereophonic sounds were then summed together for the left and right channels.

### Speech Intelligibility

Each group of 25 sentences, accompanied by the time-reserved speech noise, was transcribed by an ASR software. Two ASR software produced by different companies were used, namely, the Dragon Professional Individual (Version 16) and Meta Innovation's Dictation App, to show results for comparisons. The number of correctly transcribed key words (each group contains exactly 100 key words) was then counted to yield a speech-recognition score. Before we started the experiment with both target and interfering sound, we verified the software's performance by feeding it with clean sentences from the target pool, which was 96% correct with the first group of 25 sentences. During the experiment, the performance for the mixture of target and interference was compared before and after the spatial segregation algorithm was applied. The ASR utilizes artificial intelligence to detect speech based on a deep-learning model. Because it transcribes a monophonic speech stream (i.e., it does not benefit from stereophonic input), the left and right channels of the sound mixture were always added together before being fed into the software for both *control* and *processed* conditions. To measure the statistical significance of performance, such as the speech-recognition score, *â*, which is a percentage, we computed the confidence interval, *CI*, as

$$\mathrm{CI}=\pm 1.960∙\sqrt{\frac{\hat{a}\left(1-\hat{a}\right)}{n}}$$ (1)

(Lock et al., 2017). Here, *n* is the number of samples (*n* = 100 key words for each group of sentences). The *p* value is .05 when the coefficient 1.960 is used in Equation 1. Because the sentence-recognition task was an open set, rather than a closed set, there was no nonzero chance level. As long as the lower boundary of the 95% confidence interval (i.e., $\mathrm{CI}-\hat{a}$ ) was greater than 0, we could say that the performance was significantly higher than zero.

To examine if two scores, *a*_1_ and *a*_2_, are significantly different, a two-proportion *z* test (Lock et al., 2017) is computed as

$$z=\left(a_{1}-a_{2}\right)/\sqrt{a_{1}\left(1-a_{1}\right)/n+a_{2}\left(1-a_{2}\right)/n}$$ (2)

with the null hypothesis being *H*_0_ : *a*_1_ = *a*_2_. Since the same control performance was repeatedly compared to processed conditions, a Bonferroni correction (Dunn, 1961) was applied to adjust the significance level.

### Sound Localization Model

All simulations and signal processing were performed in MATLAB (MathWorks) at a sampling frequency of 32.1 kHz. As mentioned above, we previously developed a spiral model (Orr et al., 2023), which can be used to make quick predictions for multiple sound sources in azimuth. In that study, HRTFs from the CIPIC database (Algazi et al., 2001) were used to create virtual locations. Here, the model was instead constructed using HRTFs from the Princeton 3D3A database (Sridhar et al., 2017), which elicits more realistic spatial sensations based on our experience.

To create the basic horizontal localization model, a broadband sound of 5 s at various virtual frequencies in the frontal hemifield was presented, while elevation was set to 0. The noise signal, being binaural in the virtual acoustic space, was then transformed into the time-frequency domain by means of STFT separately for the left and right channels. The temporal window size for the STFT was 40 ms. Performing the STFT results in a series of complex numbers.

Next, a “normalization” algorithm developed by Keyrouz (2017) was applied to the STFT datapoints.

$$X_{R}\left(f,t\right)=\frac{X_{R}\left(f,t\right)}{\sqrt{{\left|X_{L}\left(f,t\right)\right|}^{2}+{\left|X_{R}\left(f,t\right)\right|}^{2}}}e^{-i\varphi_{L}}$$ (3)

**Figure 2.**
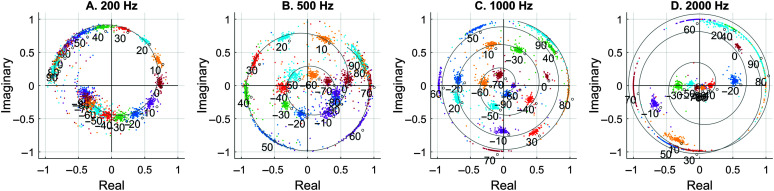
Model simulations at four representative frequencies derived with Equation 3, showing the cluster centers when a single broadband noise moves from −90° to +90° with a 10° step in the azimuth. The gray curves were fitted to approximate the spiral locations. If Equation 4 is used, the positive and negative locations switch.

Here, *X_R_* is the complex right-channel signal component at a particular frequency, including both the magnitude and the phase. *φ_L_* is the phase of the left-channel sound. The real and imaginary parts of the normalized *X_R_* are typically plotted against each other at a certain frequency. After normalization, the phase becomes the difference between the right- and left-ear phases (R–L), which is identical to the interaural phase difference (IPD) and is uniquely related to the ITD at a certain frequency. This process (Equation 3) ensures that all the datapoints reside inside the unit circle (see Figure 2) because of the denominator, while keeping the ITD and ILD cues. Specifically, if the right ear leads the left, the normalized phase becomes positive (it will move counterclockwise in the model space as the ITD increases). This is how ITD/IPD cues are preserved. Regarding ILDs, when the left ear receives a stronger signal (i.e., the source is closer to the left ear), the corresponding cluster (|*X_R_*|) lies nearer to the origin, 0. When the right ear is louder, |*X_R_*| approaches the unit circle (Orr et al., 2023).

Figure 2 displays the horizontal localization model at four representative frequencies. As can be seen in Figure 2, the cluster at each location (a particular color) generally moves in an outward spiral pattern when the horizontal location travels from the left (−90°) to the right (+90°). Briefly, the ITD cue creates the counterclockwise turning, whereas the ILD cue moves it from the center to the periphery. For more details, refer to Orr et al. (2023). At low frequencies, there are fewer turns than at high frequencies. Although the general trends are the same, there are slightly more “turns” in the current model obtained with the 3D3A database compared with the model obtained with the CIPIC database (Orr et al., 2023). This observation indicates that the exact model appearance may vary with the microphone system.

We should also point out that broadband noise was only used in creating the model locations for single sources. It is desirable because its energy covers the entire frequency range. However, broadband noise violates the sparseness property and thus cannot be segregated from speech signals using our segregation algorithm.

**Figure 3.**
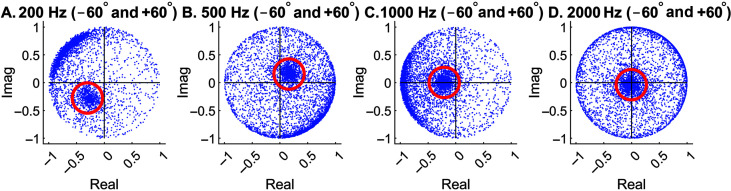
Responses in the model space after short-time Fourier transform and the normalization process elicited by two concurrent speech sources at −60° and +60°. At each frequency, the red circle marks the source to be removed or attenuated by the algorithm (time-reversed speech, +60°). For the hard mask, points inside the red circle were removed. For the soft mask, points inside the red circle were multiplied by 0.2. Imag = imaginary.

In addition, using the original equation (Equation 3) developed by Keyrouz (2017), clusters are more focused for leftmost locations and are spreading out toward the unit circle for rightmost locations. This is especially the case at high frequencies (see Figures 2C and 2D). If the unwanted sound source lies at a rightmost location, it would be difficult to remove as it does not “cluster” well. The following example (see Figure 3) shows the responses of two sound sources in the model space when the speech target was located on the left (i.e., −60°) and the interfering sound was located on the right (i.e., +60°). If Equation 3 were used, the interfering sound to be removed would have been spreading out on the unit circle (not the cluster with the red circle).

Therefore, we developed a second equation for the normalization process, as follows:

$$X_{L}\left(f,t\right)=\frac{X_{L}\left(f,t\right)}{\sqrt{{\left|X_{L}\left(f,t\right)\right|}^{2}+{\left|X_{R}\left(f,t\right)\right|}^{2}}}e^{-i\varphi_{R}}$$ (4)

It is almost the same process as Equation 3 except that L and R are flipped. With Equation 4, the interfering sound will have clusters inside the red circles (see Figure 3) and are thus easier to remove. Meanwhile, it does not matter whether the speech target forms a cluster or not for our purpose, because the inverse STFT (ISTFT) will then transform everything back to the time domain. Equation 4 was used for the entire simulation in this study, because the interfering sound was always to the right of the target location.

### Speech Segregation

As shown above, the locations of the target and interfering sound can be identified by comparing the clustering responses (see Figure 3) to the spiral model. Theoretically, the locations can be identified with a single-frequency model, which was the topic of our previous study (Orr et al., 2023). However, to remove or attenuate a relatively broad-spectrum sound, such as speech or time-reversed speech, the clusters at all frequencies must be separately identified and removed. Since we had a sampling frequency of 32.1 kHz, we performed the clustering algorithm from 25 Hz all the way to 16.0 kHz, with a frequency step of 25 Hz. This is not necessary when implementing the algorithm in real-world applications, because speech energy is mostly under 4 kHz, and typically a much lower frequency resolution would be used. For example, considering the sentences used in this study, the speech energy under 4 kHz (measured after a low-pass filter) is in the range of 91%–94% of the overall energy for the eight sentence groups.

First, the simulated target and interference mixture, both being bilateral in the virtual acoustic space, were summed together and transformed into the time-frequency domain by means of STFT. This is to mimic the situation when bilateral hearing aids receive a mixture of sound sources at both ears. Once clusters formed at each frequency, the one corresponding to the interfering sound was identified. A binary mask can be created by setting the time-frequency points inside the red circle to zero. This is called a “hard mask,” that is, 1 or 0, with 0 being complete removals. Alternatively, those points can be attenuated, instead of being removed, by creating a “soft mask.” In the present study, we set the soft mask as 1 or 0.2. Here, the value 0.2 provides a 14-dB attenuation in the signal strength. Theoretically, the soft mask cannot completely remove the unwanted source; however, if a target datapoint is misidentified as noise, keeping it in a compromised form may preserve useful information. The radius of the red circle was kept as 0.2 in this study.

Once a binary mask is created, it will multiply with the sound mixture's spectrogram. The product will then go through the ISTFT, transforming the altered sound mixture back to the time domain. Note that this is not applied to the *normalized* cluster responses shown in Figure 3. The cluster plots are simply used to identify the time-frequency points in the original STFT to be removed. In other words, there is no such thing as “inverse normalization” needed in our process.

## Results

We will first present clustering results showing how the segregation works, without needing the ASR software. Next, the software's speech-recognition scores will be presented for control (i.e., no segregation algorithm applied) and the processed conditions. For HRTFs-1 and HRTFs-2, we examined the performance with the Dragon ASR. For HRTFs-3, the Meta ASR was used as a comparison.

### Sound-Source Clustering for Different Spatial Configurations

We first examined an easy condition when speech sentences were virtually placed at −60° (i.e., 60° left to the front) and interfering sound was at +60°. Figure 3 shows the STFT responses after the normalization process, which makes all responses located inside the unit circle. Four representative model frequencies (200, 500, 1000, and 2000 Hz) are shown for demonstration purpose. In reality, speech segregation needs to be performed at every frequency in the STFT that is relevant to speech signals.

As mentioned earlier, the results were created using Normalization Equation 4. The red circles were derived from the spiral localization models at +60° (see Figure 2) and are thus considered to represent the interfering sound. In the next step, points inside the red circles were to be removed or attenuated to accomplish speech segregation from the interference. The other cluster more or less spreading on the unit circle corresponds to the speech target. If Equation 3 were used, the locations of the two clusters would have switched.

**Figure 4.**
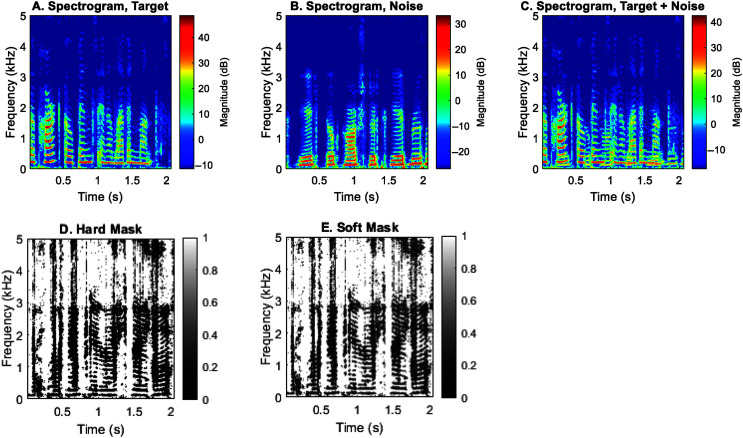
(A) The spectrogram of a target (an intelligible sentence at −60°). (B) The spectrogram of a noise (a time-reversed speech sentence at +60°). (C) The spectrogram of the target and noise presented concurrently. (D) The hard mask (1 or 0) created by identifying the cluster at the location of +60°. (E) The soft mask (1 or 0.2) created by identifying the cluster at the location of +60°.

Once clusters are formed and corresponding locations are determined, a binary mask can be generated to remove or attenuate the unwanted sound source. Figures 4A and 4B show the original spectrograms of the target and interference alone, respectively. Figure 4C shows the spectrogram when target and interference were mixed. Note that we know exactly which time-frequency point in the spectrogram (see Figure 4C) corresponds to which dot in the normalized space (see Figure 3). The hard mask (see Figure 4D) was created by setting the time-frequency points in Figure 4A corresponding to the dots inside the red circles (see Figure 3) to 0 at all frequencies. The soft mask (see Figure 4E) does the same thing except setting the value to 0.2, equivalent to a 14-dB attenuation. The rationale for using a nonzero value is to prevent abrupt removal of continuous information and, in the case of incorrectly identifying a target as noise, to reserve useful information to a certain degree.

**Figure 5.**
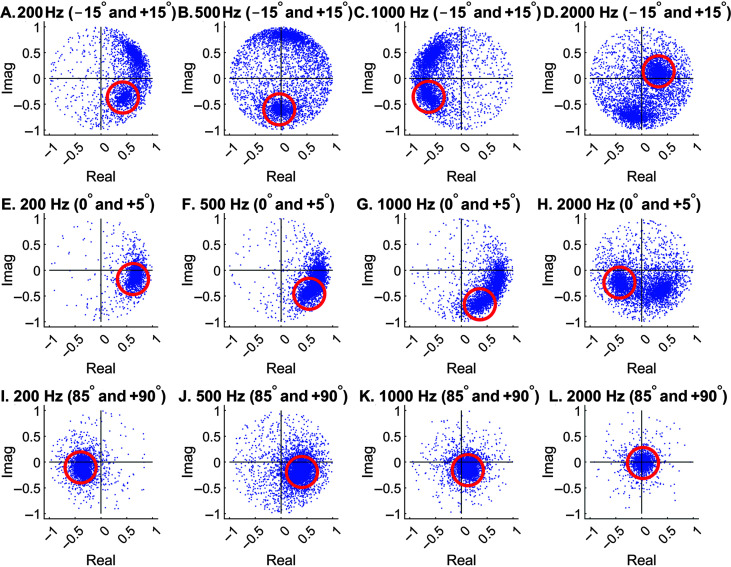
Responses in the model space after short-time Fourier transform and the normalization process elicited by two concurrent speech sources. Everything is the same as in Figure 3 except the angular separations (top, −15° and +15°; middle, 0° and +5°; bottom, +85° and +90°). Imag = imaginary.

The above example shows an easy condition when speech and interference were separated by 120°. According to the spiral model (see Figure 2), closer spatial locations may lead to closer cluster locations, depending on the frequency. Figure 5 (top row) shows the responses in the model space when speech and interference were located at −15° and +15°, respectively. At 200 Hz, since the model only has a single turn (see Figure 2A), the −15° and +15° clusters are close (see Figure 5A). They can still be apart when the model has multiple turns, such as the 500-Hz case (see Figure 5B). Most of the time, the two clusters are well separated.

When the two locations further decreased to 0° and +5° at the front, the two clusters cannot be clearly separated except at the first three frequencies (see Figure 5, middle row). Here, datapoints inside the red circles corresponding to the interference's location (+5°) were removed by comparing the responses with the spiral model (see Figure 2). This operation inevitably removed certain speech-target energy at low frequencies.

The problem gets even worse when the sound sources move to the far side, where HRTFs are inherently less separable. As can be seen in Figure 5 (bottom row), the two clusters with a spatial separation of 5° were completely indistinguishable at all four frequencies. To be consistent, we still removed the datapoints inside the red circle, which is not recommended for real-world applications.

### Speech Segregation, Hard Mask

**Figure 6.**
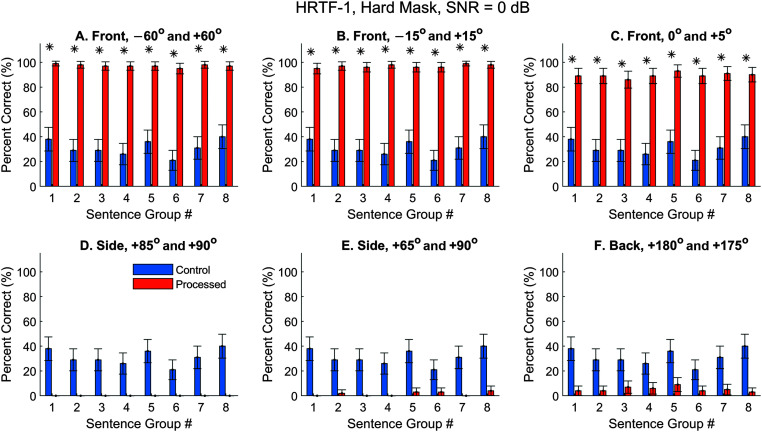
Automatic speech recognition (ASR) speech-recognition scores obtained with the hard mask using Dragon ASR and HRTFs-1 (the 1st subject in the 3D3A database). The interference noise was time-reversed speech sentences (SNR = 0 dB). Performance varied with the spatial configuration of the target (intelligible sentences) and interfering sound (time-reversed sentences). The spatial configurations of the target and interference correspond to the locations in Figure 1. (A−C) The interference and target were both located at the front with the target always being on the left side of the interference. (D−E) The interference and target were both located at the right side with the target always being more leftwards. (F) The interference and target were both located at the back with the target being at 180°. Blue, control performance when no segregation algorithm was applied. Orange, performance when the segregation algorithm had been applied to remove/attenuate the noise cluster. Error bars are 95% confidence intervals. Asterisks mark significant improvements with the segregated condition (*z* test, *p* < .05). HRTFs = head-related transfer functions.

First, the control condition was examined by feeding the speech and interfering sound directly to the ASR software. Both speech and interference had been convolved with the HRIRs (i.e., time responses of the HRTFs) to add spatial cues. The spatial configurations of the target and interference in Figure 6 correspond to the locations in Figure 1. Figure 6 (blue) shows the control performance using the Dragon ASR and HRTFs-1 obtained from the first subject in the 3D3A database. Error bars are 95% confidence intervals (Equation 1). If the lower boundary was greater than zero, we could say that the performance was significantly higher than zero.

Because the ASR transcribes monaural signals, its performance was not affected by spatial configurations of the target and interference in the control condition (see Figure 6, blue). To the ASR, the input was essentially the summation of the target sentences and the interference. Therefore, the values of the blue bars remained the same in Figures 8–10 as long as the same ASR software (i.e., Dragon) was used. However, the control performance can vary slightly when a different ASR (i.e., Meta Innovation's Dictation App) was used due to different software strategies. In Figure 6, the performance varied with the particular sentence group with an average of 31% and was generally well below 50%. For example, for the 25 target sentences in Group 1, eight sentences missed all key words, four had only one key word correct, six had only two key words correct, and six had only three key words correct. The result highlighted the masking effect of the time-reversed sentences on the speech target. We need to point out that a real person may perform differently from the two ASR software products. The reasons for using the ASR in this study will be presented in the Discussion section.

Once the clusters were formed, a binary mask can be applied to remove or attenuate the unwanted sound source. Six spatial conditions at the front, side, or back hemifields were examined. When the target and interference locations were −60° and +60° (see Figure 6A, orange), the speech-recognition performance with an average of 97% was on a par with the performance obtained with clean speech (i.e., 96%, not plotted). Of course, this yielded a highly significant improvement from the control performance for every sentence group (see Figure 6A, asterisks). Note that all significance tests have been applied with the Bonferroni correction since the same control performance (*blue*) was used six times in the comparison with different processed conditions (*orange*).

When reducing the angular separation at the front from 120° to 30° (see Figure 6B, orange), the average processed performance was still 97%, given that two clusters can still be well distinguished in the model space (see Figures 5A–5D). When the angular separation was further reduced to 5°, a small but significant decrease in the performance occurred with an average as 90% (see Figure 6C, orange). Referring back to the clustering plots (see Figures 5E–5G), the target and the interference could not be well separated at low frequencies. Consequently, the removal of interference energy would lead to undesired removal of target energy, and hence the slightly decreased performance.

However, when both sounds moved to the right side (see Figures 6D and 6E) or the back (see Figure 6F), the processed-condition performance (*orange*) was much worse. The lowest performance occurred at the side when the angular separation was 5° with an average of near 0 (see Figure 6D). This result is not surprising given that the two clusters were essentially indistinguishable under this condition (see Figures 5I–5L).

### Speech Segregation, Soft Mask

**Figure 7.**
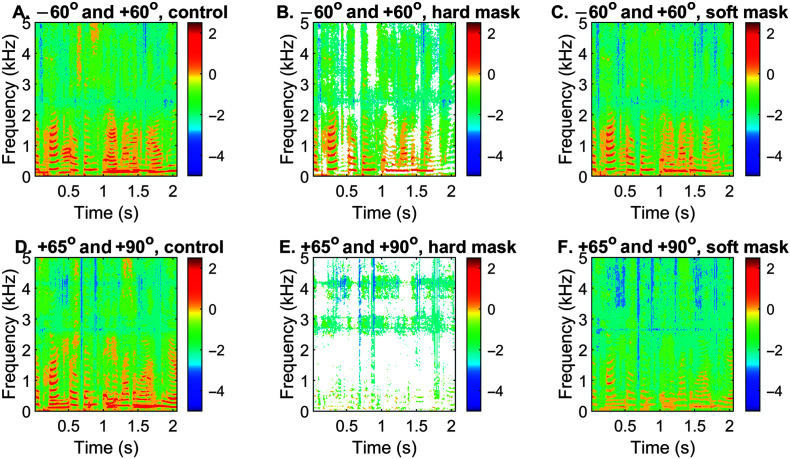
Spectrograms of a target (an intelligible sentence) and noise (a time-reversed sentence) before (left column) and after the segregation algorithm was applied by using a hard (middle column) or a soft (rightmost column) mask. The top row shows the spectrograms for the front condition with large separations. The bottom row shows the spectrograms for the side condition with only a 5° separation.

To lessen the detrimental effect when the target is collectively removed by algorithm, we explored a soft mask with which the unwanted source was only attenuated by a factor of 0.2. Figure 7 shows examples of spectrograms before (leftmost column) and after (middle and rightmost columns) the segregation algorithm was applied to the mixture of speech and interference. When the two sound sources were highly separable in the frontal hemifield (see Figure 7A), applying a binary hard mask only removed the energy of the interference (see Figure 7B). Applying a soft mask removed the energy of the interference to a lesser degree and prevented abruptness in the spectrum (see Figure 7C). In contrast, when both were at the side with only a 5° separation (see Figure 7D), applying the hard mask pretty much removed all speech-target energy (see Figure 7E), whereas the soft mask somewhat preserved the speech energy, although it also attenuated it (see Figure 7F).

**Figure 8.**
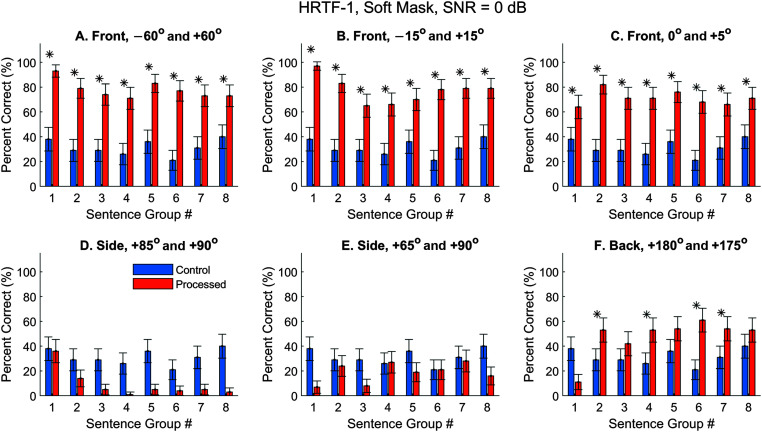
Automatic speech recognition (ASR) speech-recognition scores, soft mask, using Dragon ASR and HRTFs-1. Everything was the same as in Figure 6 except that the soft mask was applied. SNR = 0 dB. HRTFs = head-related transfer functions. Asterisks mark significant improvements with the segregated condition (*z* test, *p* < .05).

The spectrogram plots indicate that the soft mask should better preserve the target energy under difficult situations. In Figures 8A–8C, the front performances were not as high as with the hard mask (see Figures 6A–6C), with average correct rates of 78%, 76%, and 71% for the three conditions. This is expected because the interfering sound was only attenuated to 20% of the original values. For the two conditions on the right side (see Figures 8D and 8E), the processed performances were still worse than the control, but better than what was achieved with the hard mask (see Figures 6D and 6E). For the back condition with a 5° angular separation (see Figure 8F), the processed performance was significantly improved from the control for five out of eight sentence groups.

### Using Different HRTFs and/or ASR Software

**Figure 9.**
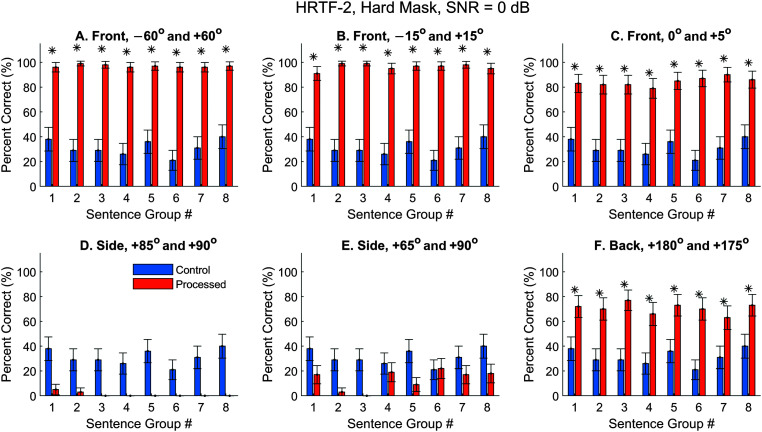
Automatic speech recognition (ASR) speech-recognition scores using Dragon ASR and HRTFs-2, hard mask. SNR = 0 dB. HRTFs = head-related transfer functions. Asterisks mark significant improvements with the segregated condition (*z* test, *p* < .05).

**Figure 10.**
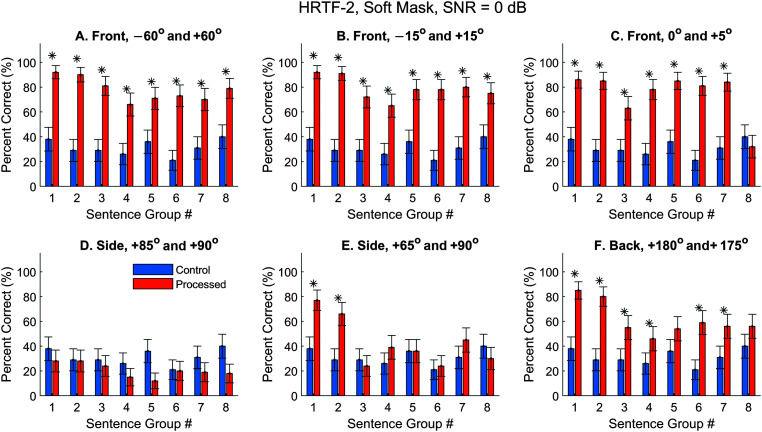
Automatic speech recognition (ASR) speech-recognition scores using Dragon ASR and HRTFs-1, soft mask. SNR = 0 dB. HRTFs = head-related transfer functions. Asterisks mark significant improvements with the segregated condition (*z* test, *p* < .05).

Because HRTFs can be highly individualized (Jiang et al., 2023) and the virtual-space technique depends on the quality of HRTFs, it is possible that some observations were only true for the HRTFs-1 we chose (from Subject 1 in the 3D3A database). First, we repeated the measurements for a different set of HRTFs (Subject 15 in the 3D3A database, randomly chosen). The control performance (blue) did not vary with the HRTFs because the ASR takes monaural input. For the processed results, observations were generally the same but slightly better. Figures 9 and 10 display the hard- and soft-mask results, respectively.

The hard-mask performance using HRTFs-2 (see Figure 9) was highly similar to the results using HRTFs-1 (see Figure 6). That is, the three frontal conditions had very high performances (see Figures 9A–9C), and the two side conditions had low performance (see Figure 9E). The biggest difference is that, for HRTFs-2, the back condition also showed high performance with an average of 71%. This is likely caused by the better qualities of HRTFs in the back hemifield obtained with this subject. When the soft mask was applied (see Figure 10), the general observations were also similar to observations with HRTFs-1 (see Figure 8), although the performance varied with the individual sentence group in a different manner.

To summarize the results for the first two sets of HRTFs, when the clusters were clearly separated in the model space, the hard mask would achieve high segregation performance, and the soft mask would yield slightly worse performance. When the clusters were not well separated, the hard mask yielded low performance, and the soft mask yielded moderate performance. In either case, the soft mask is a compromised approach that guarantees decent performance.

**Figure 11.**
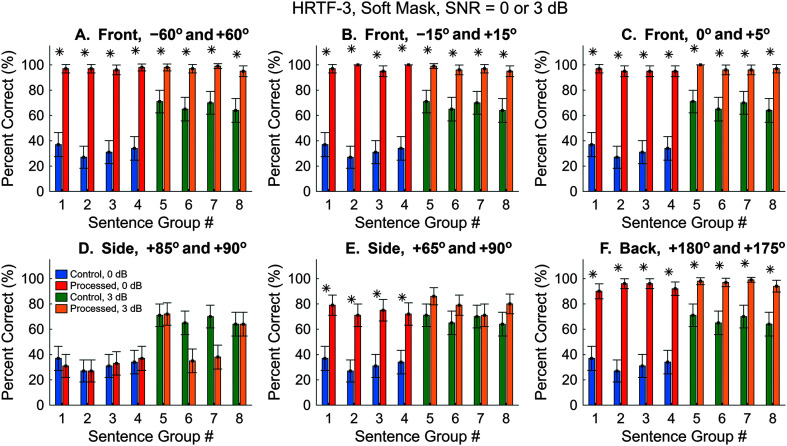
Automatic speech recognition (ASR) speech-recognition scores using Meta ASR and HRTFs-3, soft mask. Two SNR values were tested. Sentence Groups 1–4 (blue and red) had an SNR of 0 dB. Sentence Groups 5–8 (green and yellow) had an SNR of 3 dB. HRTFs = head-related transfer functions. Asterisks mark significant improvements with the segregated condition (*z* test, *p* < .05).

The above results were obtained using the Dragon Professional ASR software with an SNR of 0 dB. Next, we switched to a different software, the Meta Innovation's Dictation App, with HRTFs obtained from a third subject in the 3D3A database. We also wanted to examine if the same observations hold at higher SNR values. Figure 11 (blue and red bars) shows the control and processed scores for Sentence Groups 1–4 using the soft mask. The control performance obtained with the Meta ASR (see Figure 11, blue) was highly similar to that of the Dragon ASR (see Figures 6 and 8–10) with small distinctions. For example, the Dragon ASR showed the lowest control performance with Group 4, whereas the Meta ASR showed the lowest score with Group 2 (see Figure 11, blue). Another difference is that the Meta ASR showed higher scores with the soft mask than the Dragon ASR for all but one of the “side” conditions (see Figure 11D, red, +85° and +90°).

Next, the SNR was increased to 3 dB to examine how the algorithm works in easier listening conditions. This was realized by reducing the interference sound level by 3 dB (i.e., scaling it down to 0.707). Figure 11 (green and yellow bars) shows the control and processed scores for Sentence Groups 5–8 with an SNR of 3 dB. The control performance increased from ~30% to ~70% (green). The processed results were similar to the 0 dB performance, because the interference sound was highly attenuated by the soft mask in both conditions. Again, no benefit was observed with the 5°-separation side condition (see Figure 11D). Overall, results with the Meta ASR, the third set of HRTFs and a higher SNR were qualitatively similar to what was observed previously.

### Interference as Nonreversed Sentences

When both the target and interference were intelligible sentences with equal sound intensities (i.e., SNR = 0 dB), the ASR software cannot distinguish between the two sources since no spatial cues were utilized by the software. The transcriptions were sporadic and nonsense. For example, below are the transcribed results for the entire first group of 25 sentences with the Meta ASR.

The part of the business at 11 the white house is the old man amazed for andmother randmother wears colorful dresses barely ctor my brother lost his school is large crowded the cities is fairly crowded holiday the red vegetable so s need more toast and toast the girl loves the cream cheese horrible my baked brown taste of vegetable vegetables grow in the garden st father of my brother.

**Figure 12.**
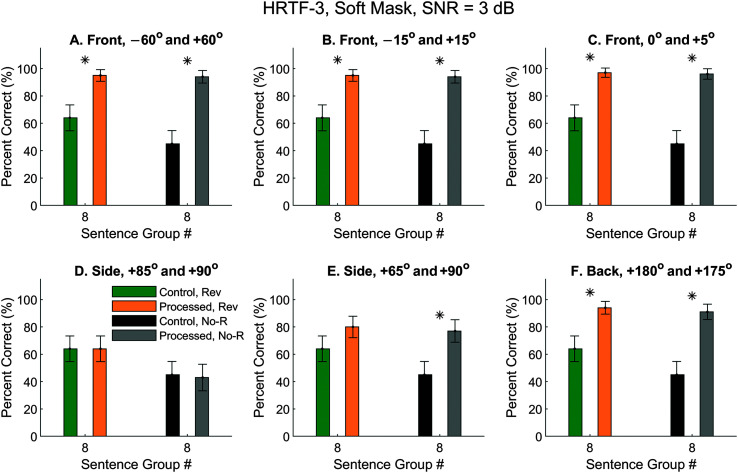
Automatic speech recognition (ASR) speech-recognition scores with nonreversed noise and a 3 dB SNR. Results were obtained with Meta ASR and HRTFs-3, using only Sentence Group 8. Green and yellow bars were control and processed results for time-reversed noise replotted from Figure 11, Group 8. Black and gray bars were control and processed results for nonreversed noise for the same set of sentences. HRTFs = head-related transfer functions. Asterisks mark significant improvements with the segregated condition (*z* test, *p* < .05).

Therefore, we used time-reversed sentences as the interference noise. However, when SNR was increased to 3 dB, the ASR can more or less focus on the louder speech. Figure 12 compared the performance of the Meta ASR with the same group of sentences (Group 8) and a SNR of 3 dB for time-reversed and nonreversed interference. The green and yellow bars are replotted from Figure 11. When the interference was also meaningful sentences, just like the target, but softer, the control performance decreased from 64% to 45% (see Figure 11, black bars). This decrease was statistically significant (*z* test, *p* = .006). Nevertheless, the processed condition showed similar performance as the time-reversed results (see Figure 12, gray bars), because, if the interference sound is highly attenuated by the algorithm, it would not matter whether the sentences were reversed or not.

## Discussion

Spatial separations between speech and interfering noise can often improve speech perception, a phenomenon called “spatial release from masking” (Saberi et al., 1991). The cocktail-party problem (Bodden, 1993; Bronkhorst, 2000; Cherry, 1953; Du et al., 2011; Haykin & Chen, 2005; Power et al., 2012; Yost, 1997) is an example when listeners are trying to understand speech in a noisy environment with multiple competing sounds and is particularly troublesome for hearing-aid users. In our previous study (Orr et al., 2023), we developed a sound-processing algorithm that can automatically decode sound locations using a simple model. Here, applying a similar strategy, we investigated an algorithm that can segregate the sound into different streams with a binary mask according to their identified locations. Combing the localization and segregation features, our algorithm can potentially provide hearing-aid users with a customized listening environment.

### The Spiral Model for Sound Localization

In the initial localization model (Orr et al., 2023), we used the CIPIC database to create virtual spatial cues. In the present study, we used the 3D3A database. The spiral model behaviors were almost identical in that the clusters spread (i.e., move further apart) and the spirality (i.e., number of turns) increases as the model frequency increases (see Figure 2). However, the model using the 3D3A database showed more turns at the same frequency than the CIPIC database. We recently performed hardware recordings in our lab by attaching a pair of binaural microphones (Sound Professionals) to a dummy head to create a real-world localization model. The spiral model shapes were again different from the simulations using the two databases, showing even fewer turns than either database. Therefore, there is not a universal spiral model for any binaural-microphone system. The exact model shape may depend on the placement of the microphone and the hardware system and should be individually calibrated.

We should also point out that, for the localization model and the segregation algorithm to work properly, the binaural microphone does not need to be deeply inserted into the ear canal to obtain traditional well-looking HRTFs, such as one that shows the midfrequency notch for vertical localization. The separation of the clusters in Figure 2 was caused only by ITDs and ILDs. In applications, it is important to make sure that the model used for identifying sound locations is created with the same recording system of the hearing device that uses the segregation algorithm.

In the previous localization study (Orr et al., 2023), after the clusters were formed, the cluster centers were identified and compared to the spiral model to derive an actual location. The present study didn't perform this type of localization per se. For example, in the −60° and +60° case, the spiral model's position for +60°, using Equation 4, was simply projected to the clusters as the red circle. In real-world applications when the user needs to first localize the sound sources before they can identify which source to be removed, the algorithm will first decide the sound locations using the K-means clustering approach (Orr et al., 2023).

Under the side condition (+85° and +90°) when only one cluster clearly formed (see Figures 5I–5L), the algorithm should decide not to remove any sound energy, because doing so will inevitably lead to worse speech intelligibility as presented in Figures 6 and 8–10. In fact, we suggest that similar decisions should be made whenever a single cluster is detected in real-world applications, just to be safe.

### General Methods for Spatial-Speech Segregation

Previous speech-segregation approaches have been constructed using a range of methods, including physiologically based models and complex machine-learning algorithms, such as convolutional neural networks (CNNs) or deep neural networks (DNNs). Most of the algorithms focus on segregation alone without providing mechanisms or measurements for localizing the sound, even when their segregation algorithms rely on sound localization cues.

Awotunde et al. (2020) used the CNN algorithm on monaural speech in background noise. Their model separated two vocalization gestures and reestimated the speech signal. They achieved high speech-intelligibility performance, for example, 93.5%. Approaches using DNN and supervised machine learning have also been explored (Bentsen et al., 2018). In that study, speech intelligibility after the segregation algorithm showed an increase of 7%–8% compared with control conditions. Although the improvements weren't large, they are valuable achievements given the difficult listening conditions (e.g., speech sentences mixed with nonstationary six-talker noise).

As mentioned in Introduction, segregation algorithms based on machine-learning models can be computationally expensive. Extensive training of the machine-learning classifiers is also critical, as the lack of available training data may impair the robustness of the models. Furthermore, due to the complexities of the CNN or DNN used in those studies, training of the model for real-time updated environments and scenarios can be time consuming, costly, and even impractical.

In terms of spatial-sound segregation, there are other physiologically based methods, for example, Roman et al. (2003). In that study, a virtual-acoustic-space approach similar to what was used in the present study created spatial sound at the ear drum. ITDs and ILDs were used to generate a binary mask. No human or ASR were recruited for speech recognition; rather, the SNR was their indicator of the segregation performance. Results were presented as relative differences (in dB) between their model and the ideal binary mask, which were close.

Chou et al. (2019) constructed a simple spiking model that converts binaural sound inputs into cortical spiking activity. Their model consists of the peripheral filter bank, a midbrain localization network, and a cortical network. In the end, the waveform of the target speech is reconstructed by a linear algorithm. In their following study (Chou et al., 2022), spatial-speech segregation was carried out using a spatially tuned neuron (STN) approach. STN's are tuned according to the best ITD and ILD for a specific angle. Spatial tuning of the algorithm is exhibited by spiking activity as a function of stimulus location. While overall performance was good, there were limitations associated with “spatial leakage,” resulting from the ambiguity of binaural cues where a binaural cue can occur for stimuli from multiple locations.

### Hard Versus Soft Mask

In this study, we extended the previous localization model (Orr et al., 2023; Figure 2) into a speech-segregation model by applying a binary mask to remove or attenuate unwanted sound sources. Speech intelligibility was measured by feeding the raw or processed virtual sound mixture into an ASR software. The localization and segregation models are based on the sparseness property of daily sound (e.g., speech and music). Because spatial cues are crucial to our model and HRTFs vary substantially with the sound location, our model performance depends highly on the general placements of the target and noise.

When the cluster of the interfering sound was widely separated from the cluster of the target, the segregation model using the hard mask achieved speech-recognition scores that were as high as the clean speech for all three sets of HRTFs (i.e., models constructed with HRTFs from three human subjects). Note that clear separations in the model space do not necessarily require large spatial separations (≥ 15°) of the sound sources. Even at the front with a 5° angular separation, the clusters may still be well separated at many frequencies (see Figures 5E–5H) because humans have sharp horizontal acuity at the front median plane.

Our best segregation results may have outperformed human performance. For all but the last condition, the speech target and time-reversed sentences were equal in their sound intensity, creating a 0 dB SNR. Yet, the best individual intelligibility score reached 98% after applying the hard mask. In this near-perfect case, using the soft mask (a 20% attenuation) will inevitably decrease speech intelligibility due to residual energy of the noise. This is observed with model performance using both sets of HRTFs.

The observations changed when both the target and interference moved to the side or back. With a 5° angular separation at the back, the sound mixture processed with the hard mask for HRTFs-1 was barely above zero. (To be significantly above zero, the lower boundary of the confidence interval needs to be higher than zero.) Switching to the soft mask significantly increased the intelligibility for 5/8 sentence groups (see Figure 8F). This is not surprising since the back hemifield is more difficult for humans and machines to localize than the front (Aggius-Vella et al., 2017; Makous & Middlebrooks, 1990; Oldfield & Parker, 1984), and, hence, the speech segregation. The story was somewhat different for HRTFs-2. This set of HRTFs had a high performance at the back with hard-mask processed sound (see Figure 9F), although not as high as the front ones. It is probably due to higher quality of measured HRTFs. Switching to a soft mask actually decreased the performance, which was still significantly higher than control for all eight-sentence groups. The explanation is the same as for the frontal locations.

In the present study, the side performance for 5° or 15° separations was worse even than the control with the hard mask. It was not surprising given that the hard mask with a 5° separation at the right side almost entirely removed the energy of both target and interference (see Figure 7E). The cluster plots show that only a single cluster can be identified (see Figures 5I–5L). Human studies indicated that localization errors at the back are generally larger than the side (Makous & Middlebrooks, 1990; Oldfield & Parker, 1984), we could not understand why our back performance was better than the side performance. One possible explanation is the “front-back confusion” (Carlini et al., 2024) commonly experienced by human listeners in sound localization. In the present study, we did not test target and interference locations that may sit on the confusion cone. In that case, the ITD cue would be identical, and the ILD and spectral cues may vary for the front and back due to the pinnae's shadow effect. Our model performance will certainly be affected by front–back confusion if we remove the datapoints inside the red circle at every frequency. Therefore, a rule should be made clear that if only one cluster is identified and few datapoints exist outside the cluster (e.g., Figures 5I–5L) at a certain frequency, the cluster shall not be removed or attenuated.

In addition, we would like to point out that when we informally listened to the segregated speech, the quality was always good for the front locations. The quality of the speech was not good for the side locations due to significant portions of target energy being removed together with the noise. The quality of the speech was acceptable for the back locations, although not as good as that of the front locations.

### ASR and Auditory-Scene Analysis

In this study, we used the Dragon ASR, rather than human subjects, to examine the effect of masking and segregation of the target speech for several reasons. First, there were many experimental conditions to be tested and compared. When the control (see Figures 6 and 8–10, blue) was included, there were a total of seven conditions for each of the four bar plots. Each condition consisted of eight groups of sentences, creating a total of 200 sentences to be graded. A single subject would have performed a total of 200 ∗ 7 ∗ 4 = 5600 sentences. But more importantly, we cannot repeat meaningful sentences to the same human subject. If they had heard a relatively clean sentence in the frontal conditions, they would have remembered it even when they could barely hear it later. In other words, each new condition would require a brand-new set of sentences to be presented to the same subject. Last but not least, for intelligible sentences, humans are capable of guessing completely inaudible words based on the context (Gianakas & Winn, 2025). The ASR's ability to do so will be limited and dependent on its training. Therefore, using the ASR may provide a more stringent examination on energetic masking.

That said, the argument against perceptual testing was incomplete. A counterbalanced design with multiple listeners could be a feasible option. In future studies we will test the segregation algorithm in a hardware setting with human subjects. We may use a closed-set discrimination task rather than an open-set sentence task to compare across conditions.

As mentioned in Introduction, certain ASR algorithms already contain monaural speech segregation from noise, such as using models for auditory scene analysis (Cooke et al., 2010; Cooke & Ellis, 2001; Elhilali & Shamma, 2006; Shamma et al., 2011; Shao et al., 2010). Therefore, results presented here may have reflected certain efforts of the ASR's built-in properties. Nevertheless, consistent improvements of the ASR performance after the segregation process compared with the control performance using the same ASR serves as a validation of the segregation algorithm we created.

### Limitations of the Present Study

There are several limitations with the study caused by experimental designs. First, all speech-recognition results were obtained with ASR software, rather than human listeners. Even though previous studies have shown > 90% similarities between the two (Karbasi & Kolossa, 2022; Schädler et al., 2015), there are still distinct differences between human and ASR, as well as variations across different ASR programs. For example, human listeners experience both energetic and informational masking with meaningful sentences (Brungart, 2001; Kidd et al., 2016). The present study used time-reversed sentences as the interference noise; therefore, no informational masking was involved. We decided to reverse the noise because, when trying with equal intensities of two simultaneous sentences, the ASR was incapable of providing meaningful results. However, we did show that when the SNR was increased to 3 dB, the ASR may focus on the louder target, though to a lesser degree without the segregation algorithm (see Figure 12, black bars).

Human listeners can also guess individual words in meaningful sentences (Salasoo & Pisoni, 1985). When we selected the two ASR products used in this study (i.e., Dragon and Meta), we avoided products that can be overtrained. For example, latest internet-based ASR products may find the correct answers from sources other than the audio files we provided, given that the sentences used in this study have been created and published a decade ago (Calandruccio & Smiljanic, 2012). Either way, the ASR results presented here only examined energetic masking and are not supposed to represent human performance. No complete conclusions can be made until further studies are performed with human listeners, although the ASR performance did show significant improvements with the speech-segregation algorithm in the context of energetic masking.

Second, the segregation algorithm inevitably imposes more computational demands. As mentioned earlier, since 91%–94% of the speech energy for the sentences used in this study was contained under 4 kHz, it is not necessary to perform the clustering algorithm up to 16 kHz. Nevertheless, we are unsure of the exact processing delay that would occur in a hearing-aid setting. We are uncertain if the processing speed can align with the strict requirements of real-time application to avoid distortions of perceived sound quality.

Third, prior knowledge of target or noise locations is required before the interference can be removed or attenuated. Although our localization algorithm (Orr et al., 2023) can identify horizontal locations for concurrent sound sources once the clusters are formed, it does not indicate which one is the target. There are analyses such as modulation-based voice activity detection (Kanai & Unoki, 2012) that can be used to separate speech from nonspeech sound, but they may not work in a multitalker scenario when “noise” is also speech. In addition, this type of analysis may add further processing delays to the real-time hearing device. Therefore, users of our segregation algorithm will need to make their own judgments on which sound sources to be kept or removed.

Last, this study only examined three sets of HRTFs with a small number of spatial configurations (i.e., six). Out of the six conditions, we have found discrepancies between what was observed and what was expected. For example, human studies showed that localization errors at the back are generally larger than the side. It is unclear why our algorithm based on HRTFs failed to improve the side performance but worked much better for the back. As indicated by the spectrograms (see Figure 7), replacing the ASR with human listeners for the side condition is unlikely to solve the problem, as most of the target energy has been removed. Future work should include a wider variety of pinna shapes/sizes and explore better ways of modifying the segregation algorithm. We should also examine the effect of room reverberations, which have already demonstrated harmful results in our localization study (Orr et al., 2023).

## Conclusions

This study extended a previously established sound-localization model into a spatial-sound segregation model, based on daily sound's sparseness properties. A normalization process after the STFT confines all the datapoints inside the unit circle, and the clusters move in a spiral manner with horizontal sound locations. When the speech target and interfering sound were both located at the front, applying a binary hard mask can almost perfectly remove the energy of the interfering sound. However, when clusters of the speech and noise in the model space were close or indistinguishable, the hard mask removed the energy of both speech and noise, leading to worse performance of the ASR than the control. Applying a soft mask with a 20%-attenuation strength (i.e., −14 dB) showed compromised but decent performance for all the conditions and is thus recommended. Overall, this study presents a novel methodology for spatial-sound segregation that has potential of improving spatial hearing for hearing-aid users with simple and efficient algorithms.

## Data Availability Statement

MATLAB algorithms and ASR-transcribed results can be found at the following online data repository: https://doi.org/10.7910/DVN/ZIYVFZ.
